# Traffic monitoring research based on roadside LiDAR-camera target-free wide-area collaborative calibration

**DOI:** 10.1371/journal.pone.0356394

**Published:** 2026-08-14

**Authors:** Shuang Shi

**Affiliations:** Faculty of Intelligent Transportation, Anhui Sanlian University, Hefei, Anhui, China; Southwest Jiaotong University, CHINA

## Abstract

Accurate LiDAR-camera extrinsic calibration is fundamental to roadside multi-sensor fusion for traffic monitoring. Traditional calibration methods usually depend on calibration targets and manual operation, which limits their applicability in large-scale roadside deployments. To overcome this limitation, this paper proposes a target-free wide-area calibration method for roadside LiDAR-camera systems. Instead of relying on artificial markers, the proposed method estimates extrinsic parameters directly from natural traffic scenes. It first extracts complementary geometric and structural features from point clouds and images, and then establishes cross-modal correspondences through a confidence-guided coarse-to-fine matching strategy. To improve calibration stability in dynamic roadside environments, temporal consistency is further introduced into the optimization process together with reprojection constraints. Experiments on a self-built roadside dataset demonstrate that the proposed method achieves an average rotation error of 0.185° and an average translation error of 2.36 cm. Compared with representative target-free methods, it provides higher calibration accuracy while preserving practical computational efficiency. The method also shows good robustness under challenging conditions such as low illumination, occlusion, and dense traffic flow, indicating its potential for real-world roadside deployment.

## 1. Introduction

The rapid development of intelligent transportation systems has driven road perception technology toward multi-modal fusion. As an important component of vehicle-road cooperative systems, roadside sensors can provide global traffic information beyond the perception range of individual vehicles, effectively compensating for the limitations of vehicle-mounted sensors in blind spots and occlusion scenarios. The fusion sensing scheme of lidar and camera has attracted extensive attention because of its complementary characteristics – lidar can provide accurate three-dimensional spatial position information, while camera can capture rich texture and semantic features. The premise of this heterogeneous sensor fusion is to obtain a high-precision external parameter calibration matrix between the two, which describes the rigid body transformation relationship between the two sensor coordinate systems, including rotation and translation parameters. The roadside monitoring scene has the characteristics of high sensor installation position, wide coverage, long target distance and so on, which puts forward higher requirements for the adaptability and robustness of the calibration method.

Traditional LiDAR-camera extrinsic calibration methods usually rely on specific calibration targets and offline operation in controlled environments, which limits their applicability in large-scale roadside monitoring systems [1]. Target-free calibration methods have therefore attracted increasing attention, and their core idea is to establish cross-modal correspondences by using geometric, semantic, or learned features in natural scenes [2]. Early target-free methods mainly rely on manually designed geometric primitives, such as line features, road boundaries, structural edges, and planar constraints. These methods reduce the dependence on artificial calibration boards, but their performance is easily affected by occlusion, sparse point clouds, uneven feature distribution, and noise in complex traffic scenes [3]. Pixel-level targetless self-calibration further improves the use of natural scene edges and can achieve accurate LiDAR-camera alignment without checkerboards [4]. General single-shot targetless calibration toolboxes also improve automation by estimating 2D–3D correspondences from naturally observed structures and refining the transformation through cross-modal registration [5].

With the development of deep neural networks, learning-based calibration methods have improved feature representation, cross-modal association, and calibration parameter estimation [6]. Representative DNN-based methods can be divided into two main categories. The first category directly or semi-directly estimates calibration deviation from image and LiDAR representations. For example, LCCNet constructs a cost-volume network between RGB images and projected LiDAR depth maps to estimate extrinsic calibration errors in an online self-calibration manner [7]. Transformer-based calibration networks further enhance cross-modal feature aggregation and correlation modeling; CalibFormer aggregates multi-level image and LiDAR features and uses multi-head correlation and Transformer modules for automatic LiDAR-camera calibration [8]. In roadside scenarios, some studies have designed Transformer-based calibration networks according to the specific installation height, viewing angle, and deployment geometry of traffic monitoring sensors [9]. The second category focuses on DNN-based feature extraction and matching, where dense or coarse-to-fine point–pixel correspondences are established before pose optimization. Recent targetless online frameworks also explore object-level common feature discrimination, iterative refinement, and attention-based post-refinement to improve correspondence reliability in urban traffic scenes [10].

Existing studies show that targetless LiDAR-camera calibration is evolving from single-frame parameter regression toward hybrid frameworks that combine deep feature learning, explicit cross-modal matching, and geometric optimization. In roadside wide-view monitoring, existing targetless calibration methods can also be applied, but the spatial distribution and reliability of natural correspondences become more uneven due to long observation distance, edge-region projection instability, occlusion, illumination variation, and dense traffic flow. The proposed method is positioned within this hybrid target-free calibration direction and focuses on improving correspondence coverage and optimization stability under these roadside deployment conditions. Compared with direct regression methods, it explicitly establishes point–pixel correspondences from natural roadside structures. Compared with general image-to-point cloud registration methods, it introduces region-aware correspondence selection, confidence-guided coarse-to-fine matching, and spatiotemporal collaborative optimization to reduce the influence of locally dense or unreliable matches in fixed roadside monitoring scenes.

To address the above roadside deployment characteristics, this paper proposes a target-free LiDAR-camera calibration method for roadside wide-view traffic monitoring. The method treats wide-view deployment as a condition associated with uneven feature distribution and varying correspondence reliability. The main technical components include three aspects. First, a hierarchical feature extraction strategy is designed to combine local geometric descriptors with global semantic information, improving the representation of both near-field structured regions and far-field sparse regions. Second, a confidence-guided coarse-to-fine matching mechanism with region-aware correspondence selection is adopted to obtain reliable and spatially distributed point–pixel correspondences from natural roadside structures. Third, a spatiotemporal collaborative optimization framework is constructed by integrating multi-frame observations and overlap-region consistency, which improves the stability of extrinsic parameter estimation in fixed roadside monitoring scenarios. The method provides a spatial alignment basis for roadside multi-sensor traffic perception under wide-view deployment conditions.

The rest of this paper is organized as follows. Section 2 introduces the proposed method, including the calibration model, feature extraction, matching strategy, and optimization framework. Section 3 presents the experimental setup and results. Section 4 discusses the method’s performance, application value, and limitations. Section 5 concludes the paper and suggests future research directions.

## 2. Methods

Roadside LiDAR and camera target-free wide-area collaborative calibration involves core technical aspects such as cross-modal data registration, feature correspondence establishment, and extrinsic parameter optimization. This chapter starts from problem modeling, elaborates on the design principles of target-free feature extraction and matching algorithms, constructs a wide-area collaborative calibration optimization model, and provides experimental scheme and algorithm implementation details.

### 2.1. Problem description and mathematical modeling

The essence of roadside LiDAR-camera calibration is to determine the rigid body transformation relationship between two heterogeneous sensor coordinate systems. Let the LiDAR coordinate system be L, and the camera coordinate system be C. The goal of extrinsic calibration is to estimate the rotation matrix R∈SO(3) and translation vector $𝐭\in R^{\text{3}}$ from L to C [11]. For any three-dimensional point $P_{L}=[X_{L},Y_{L},Z_{L}{]}^{T}$ in the LiDAR coordinate system, its representation $P_{C}$ in the camera coordinate system can be obtained through homogeneous transformation:


$$\begin{array}{c} P_{C}=RP_{L}+t \end{array}$$ (1)

where R represents a 3 × 3 orthogonal rotation matrix, and t represents a three-dimensional translation vector. To project a three-dimensional point in the camera coordinate system onto the image plane, the camera intrinsic matrix $K_{c}$ must be combined. Let the intrinsic matrix be:


$$\begin{array}{c} K_{c}=[\begin{array}{ccccccc} f_{x} & \text{0} & c_{x}\;\text{0} & f_{y} & c_{y}\;\text{0} & \text{0} & \text{1} \end{array}] \end{array}$$ (2)

where $f_{x}$ and $f_{y}$ are the focal lengths in the horizontal and vertical directions respectively, and $c_{x}$ and $c_{y}$ are the image principal point coordinates. The process of projecting the three-dimensional point $P_{C}$ to pixel coordinates $p=[u,v{]}^{T}$ can be expressed as:


$$\begin{array}{c} s[\begin{array}{c} u\;v\;\text{1} \end{array}]=K[R|t][\begin{array}{c} X_{L}\;Y_{L}\;Z_{L}\;\text{1} \end{array}] \end{array}$$ (3)

where s is the depth scale factor. Roadside traffic monitoring scenarios have several significant characteristics: fixed sensor installation positions, wide monitoring ranges, lack of artificial calibration objects in the scene, and continuous traffic flow movement [12]. These characteristics make traditional calibration board-based methods difficult to apply, necessitating the development of target-free automatic calibration technology.

This paper formalizes the calibration problem as a joint optimization framework. Given a LiDAR point cloud set $P=P_{i},\;i={\text{1}}^{N}$ *and an image feature set* $F=f_{j},\;j={\text{1}}^{M}$, the calibration objective function is defined as minimizing the cross-modal reprojection error:


$$\begin{array}{c} E(R,t)=\sum_{(i,j)\in M}\rho \left(|\pi (RP_{i}+t)-p_{j}{|}_{\text{2}}\right) \end{array}$$ (4)

where M represents the set of established cross-modal correspondences, π(·)is the camera projection function, and ρ(·)is a robust kernel function (such as Huber loss) to suppress the influence of outliers. The solution to this optimization problem depends on accurate feature extraction and reliable correspondence establishment.

**Fig 1:**
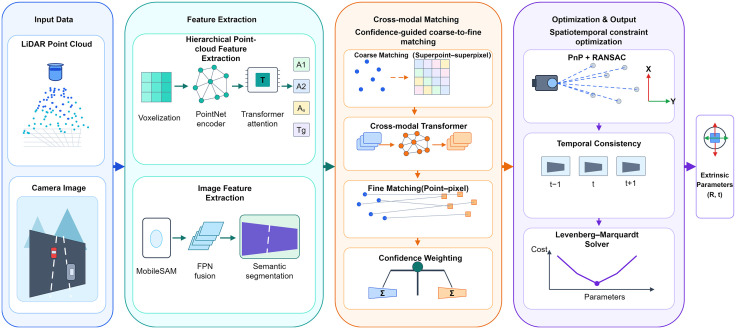
Overall Framework of Roadside LiDAR-Camera Target-free Wide-area Collaborative Calibration Method.

As shown in Fig 1, the overall process of this method includes three core modules: the hierarchical feature extraction module extracts multi-scale geometric and semantic features from LIDAR point cloud and camera image respectively; The confidence degree guides the matching module to establish the cross modal correspondence in the unified representation space; The space-time constraint optimization module fuses the multi frame observation information to solve the optimal external parameters.

### 2.2. Target-free feature extraction and matching algorithm

The core of the target free calibration method is to automatically detect and match stable cross modal features from natural scenes [13]. In roadside traffic scenes, structural elements such as road markings, lane boundaries, curbs, and light poles provide rich geometric clues for feature extraction.

The sparsity and disorder of 3D point clouds pose a challenge to feature learning [14]. This paper adopts a hierarchical feature encoding strategy that integrates local geometric description with global context information. Point clouds are first preprocessed through voxelization, discretizing continuous space into regular grids:


$$\begin{array}{c} V(i,j,k)=P_{l}|P_{l}\in \text{Voxel}(i,j,k) \end{array}$$ (5)

where V(i,j,k) represents the set of points contained in the voxel indexed by (i,j,k). Point cloud features within voxels are aggregated through a PointNet encoder [15]:


$$\begin{array}{c} f_{v}=\text{MaxPool}(\text{MLP}(P_{l}-\bar{P}l\in V)) \end{array}$$ (6)

where $\bar{P}$ is the centroid of points within the voxel, MLP(·)represents a multi-layer perceptron, and MaxPool(·) performs symmetric aggregation to ensure permutation invariance.

To capture long-range dependencies, a Transformer structure is introduced for global feature enhancement [16]. Let the voxel feature sequence be ${𝐗}_{v}\in {\text{R}}^{N_{v}\times d}$, where $N_{v}$ is the number of valid voxels and d is the feature dimension. Each row of ${𝐗}_{v}$ represents the feature descriptor of one voxel after local PointNet encoding. The query, key, and value matrices are generated by linear projections of ${𝐗}_{v}$:


$$\begin{array}{c} 𝐐={𝐗}_{v}{𝐖}_{Q},\;{𝐊}_{\text{att}}={𝐗}_{v}{𝐖}_{K},\;{𝐕}_{\text{att}}={𝐗}_{v}{𝐖}_{V} \end{array}$$ (7)

where ${𝐖}_{Q}$, ${𝐖}_{K}$, and ${𝐖}_{V}$ are learnable projection matrices. Here, 𝐐 is used to query relevant contextual information from other voxels, ${𝐊}_{\text{att}}$ represents the key matrix used to measure attention similarity, and ${𝐕}_{\text{att}}$ contains the voxel features to be aggregated. To avoid confusion with the camera intrinsic matrix 𝐊, the key matrix in the attention module is denoted as ${𝐊}_{\text{att}}$. The self-attention mechanism is computed as follows:


$$\begin{array}{c} \text{Attention}(𝐐,{𝐊}_{\text{att}},{𝐕}_{\text{att}})=\text{softmax}\left(\frac{𝐐{𝐊}_{\text{att}}^{\text{T}}}{\sqrt{d_{k}}}\right){𝐕}_{\text{att}} \end{array}$$ (8)

where $d_{k}$ is the dimension of the key vectors. By stacking multi-head attention layers and feedforward networks, the point cloud encoder outputs feature representations with global perception capabilities.

The image branch employs a lightweight segmentation-based network to extract dense pixel-level features from structured regions in roadside scenes [17]. Considering the real-time requirements of roadside deployment, a lightweight visual encoder is adopted to reduce computational overhead while maintaining effective feature representation capability [18]. The resulting multi-scale image features are further fused through a feature pyramid network (FPN) to generate a unified dense feature map for subsequent cross-modal matching.

Semantic segmentation results are used to identify structured regions such as road markings and lane boundaries. Let the segmentation mask be ${\text{M}}_{\text{sem}}$, and let conf(u,v) denote the confidence score of pixel (u,v). Effective image feature points are selected according to the semantic category and confidence threshold:


$$\begin{array}{c} {\text{S}}_{\text{img}}=\left\{(\text{u},\text{v})|{\text{M}}_{\text{sem}}(\text{u},\text{v})\in {\text{C}}_{\text{valid}},\text{conf}(\text{u},\text{v})>\tau_{\text{c}}\right\} \end{array}$$ (9)

where ${\text{C}}_{\text{valid}}$ is a predefined set of valid categories, such as road markings and curbs, and $\tau_{\text{c}}$ is the confidence threshold.

The outputs of the hierarchical feature extraction module are denoted as $O_{\text{feat}}=\{{\text{F}}_{\text{P}},{\text{F}}_{\text{I}},{\text{M}}_{\text{sem}},{\text{S}}_{\text{img}}\}$, where ${\text{F}}_{\text{P}}$ represents the encoded point-cloud feature set, ${\text{F}}_{\text{I}}$ represents the dense image feature map, ${\text{M}}_{\text{sem}}$ is the semantic segmentation mask, and ${\text{S}}_{\text{img}}$ is the set of valid image feature points selected by semantic category and confidence threshold. These outputs are used as the input of the confidence-guided matching module. Specifically, ${\text{F}}_{\text{P}}$ and ${\text{F}}_{\text{I}}$ are projected into a unified representation space to compute cross-modal similarity, while ${\text{M}}_{\text{sem}}$ and ${\text{S}}_{\text{img}}$ restrict the matching candidates to structured roadside regions. This shared-variable design ensures that the semantic filtering results obtained in the feature extraction stage are directly inherited by the subsequent coarse-to-fine correspondence estimation.

The confidence-guided matching module takes $O_{\text{feat}}$ as input and establishes cross-modal correspondences in a unified representation space. The encoded point-cloud features ${\text{F}}_{\text{P}}$ provide geometric descriptors for superpoints, while the image features ${\text{F}}_{\text{I}}$ provide visual-semantic descriptors for superpixels. The segmentation mask ${\text{M}}_{\text{sem}}$ and the valid feature set ${\text{S}}_{\text{img}}$ are used to filter image-side candidates before matching, so that correspondences are preferentially generated from stable roadside structures. Since images and point clouds have fundamental differences in data structure, information density, and semantic expression, the matching process adopts a progressive coarse-to-fine strategy to reduce ambiguity [19]. This paper draws on CoFiI2P’s coarse-to-fine matching strategy [20] to achieve progressive correspondence estimation in a unified representation space.

**Fig 2:**
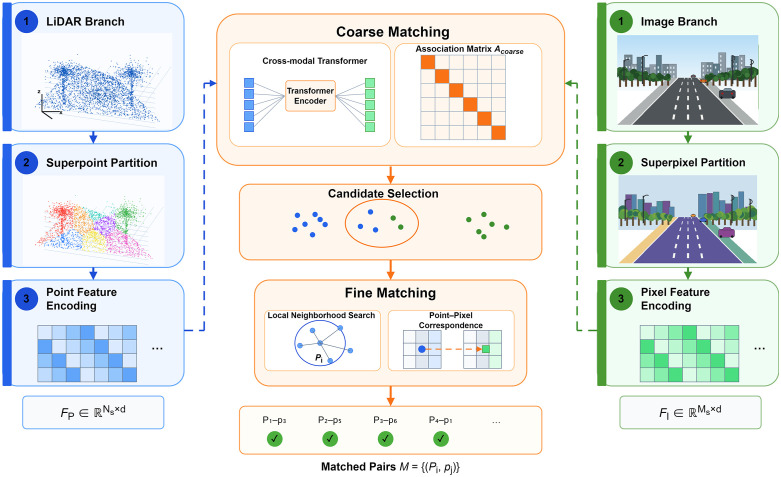
Cross-modal Coarse-to-fine Feature Matching Process.


As shown in Fig 2, the matching process is divided into two stages. In the coarse matching stage, point clouds and images are divided into superpoints and superpixels, capturing global similarity distributions through a cross-modal Transformer:

$$\begin{array}{c} A_{coarse}=\text{softmax}\left(\frac{F_{P}F_{I}^{T}}{\sqrt{d}}\right) \end{array}$$ (10)

where ${\text{F}}_{\text{P}}\in R^{{\text{N}}_{\text{s}}\times \text{d}}$ and ${\text{F}}_{\text{I}}\in R^{{\text{M}}_{\text{s}}\times \text{d}}$ denote the superpoint and superpixel feature matrices, respectively. ${\text{N}}_{\text{s}}$ and ${\text{M}}_{\text{s}}$ are the numbers of superpoints and superpixels, respectively, d is the feature dimension, and ${\text{A}}_{\text{coarse}}\in R^{{\text{N}}_{\text{s}}\times {\text{M}}_{\text{s}}}$ represents the coarse-grained association matrix. Based on this association matrix, high-confidence candidate pairs are selected, and fine-grained matching is then performed within local neighborhoods to obtain a point-level correspondence set M.

For roadside wide-view scenes, the candidate correspondences are further processed using a region-aware selection strategy. The image plane is divided into several spatial regions, and high-confidence correspondences are retained from each valid region. This prevents pose estimation from being dominated by locally dense correspondences in the near-field or image-center area and preserves useful constraints from far-field and edge regions. When a region contains insufficient valid correspondences, neighboring regions with similar semantic categories are used to supplement the candidate set. The resulting correspondence set maintains a more balanced spatial distribution and provides stable input for subsequent pose estimation.

To improve matching robustness, a confidence weighting mechanism is introduced. The confidence of each match $\left({\text{P}}_{\text{i}},{\text{p}}_{\text{j}}\right)$ is defined as:


$$\begin{array}{c} {\text{w}}_{\text{ij}}=\sigma \left({\text{f}}_{\text{i}}^{\text{P}⊤}\text{W}{\text{f}}_{\text{j}}^{\text{I}}\right) \end{array}$$ (11)

where $\text{W}\in R^{\text{d}\times \text{d}}$ is a learnable bilinear transformation matrix, ${\text{f}}_{\text{i}}^{\text{P}}$ and ${\text{f}}_{\text{j}}^{\text{I}}$ denote the point-cloud and image feature descriptors, respectively, and σ(·) is the Sigmoid function. The matrix W is trained jointly with the cross-modal feature encoders during the pre-training stage. Specifically, candidate point–pixel pairs are labeled as positive or negative according to their reprojection distance under the reference extrinsic parameters. A pair is regarded as positive if the reprojection distance is smaller than a predefined threshold $\tau_{\text{m}}$, and negative otherwise.

The learned confidence score ${\text{w}}_{\text{ij}}$ is then retained together with each candidate correspondence and passed to the pose estimation stage. In the subsequent optimization, ${\text{w}}_{\text{ij}}$ is used as the weight of the corresponding reprojection residual. Specifically, the residual of a matched pair is defined as ${\text{r}}_{\text{ij}}=\pi (\text{R}{\text{P}}_{\text{i}}+\text{t})-{\text{p}}_{\text{j}}$, and its weighted contribution is expressed as ${\text{w}}_{\text{ij}}\rho (∥{\text{r}}_{\text{ij}}{∥}_{\text{2}}^{\text{2}})$, where ρ(·) denotes the robust kernel function. Therefore, correspondences with larger confidence scores have stronger influence on the estimation of R and t, whereas low-confidence correspondences are suppressed. In the LM/Ceres implementation, this is equivalently realized by multiplying each residual by $\sqrt{{\text{w}}_{\text{ij}}}$ before constructing the least-squares problem.

The confidence branch is supervised by a binary cross-entropy loss:


$$\begin{array}{c} L_{\text{conf}}=-\frac{\text{1}}{\left|\Omega \right|}\sum_{(\text{i},\text{j})\in \Omega }\left[{\text{y}}_{\text{ij}}\text{log}{\text{w}}_{\text{ij}}+(\text{1}-{\text{y}}_{\text{ij}})\text{log}(\text{1}-{\text{w}}_{\text{ij}})\right]\; \end{array}$$ (12)

where Ω is the set of sampled candidate correspondences and ${\text{y}}_{\text{ij}}\in \{\text{0},\text{1}\}$ is the correspondence label. During training, W is updated through backpropagation together with the feature encoders. During inference, the learned W is used to assign higher weights to reliable correspondences and suppress mismatched pairs before PnP-RANSAC and spatiotemporal optimization.

Table 1 summarizes the comparison of feature extraction strategies between the proposed method and existing representative methods.

**Table 1:** Comparison of feature extraction and matching methods.

Method	Point Cloud Features	Image Features	Matching Strategy	Target-free
CorrI2P [19]	PointNet++	ResNet	Dense Correspondence	Yes
CoFiI2P [20]	PAConv	Transformer	Coarse-to-fine	Yes
CalibNet [21]	VoxelNet	CNN	Regression	Yes
Proposed Method	Voxel+Transformer	MobileSAM + FPN	Confidence-guided Coarse-to-fine	Yes

### 2.3. Wide-area collaborative calibration optimization model

In roadside wide-view deployment, existing target-free calibration methods can be applied, while the reliability and spatial distribution of natural correspondences are affected by long observation distance, uneven feature density, edge-region projection instability, and occlusion [22]. To improve calibration stability under these deployment conditions, a roadside-specific collaborative optimization framework is constructed by coupling confidence-aware reprojection constraints, fixed-sensor temporal stability, and overlap-region consistency. The geometric term is derived from confidence-guided natural-scene correspondences extracted from multiple roadside structures, including lane markings, curbs, poles, and vehicle contours. The temporal term is formulated on the extrinsic parameters over a short observation window, reflecting the physical installation condition that roadside LiDAR and camera units remain stationary while traffic participants change over time. For multi-sensor coverage, co-visible features in overlapping fields of view provide cross-sensor consistency between adjacent LiDAR-camera pairs. This architecture links feature reliability, temporal stability, and inter-sensor consistency within a unified calibration objective, thereby improving convergence stability under roadside wide-view deployment conditions.

Based on the established cross-modal correspondences, the PnP (Perspective-n-Point) algorithm is employed to solve for initial extrinsic parameters [23]. Given n groups of 3D-2D point pairs $\{({\text{P}}_{\text{i}},{\text{p}}_{\text{i}}){\}}_{\text{i}=\text{1}}^{\text{n}}$, PnP estimates camera pose by minimizing reprojection error. To enhance robustness to outliers, a RANSAC framework is embedded for iterative screening [24]:


$$\begin{array}{c} ({\text{R}}^{*},{\text{t}}^{*})=\text{arg}\underset{\text{R},\text{t}}{\text{min}}\sum_{\text{i}\in \text{I}}∥\pi (\text{R}{\text{P}}_{\text{i}}+\text{t})-{\text{p}}_{\text{i}}{∥}_{\text{2}}^{\text{2}} \end{array}$$ (13)

where I is the inlier set identified by RANSAC. The combination of PnP+RANSAC can still obtain reliable initial solutions in scenarios with high outlier ratios [25].

The dynamic characteristics of traffic scenes provide temporal redundancy information for calibration. Extrinsic parameters between consecutive frames should remain consistent, and this prior can be incorporated into the optimization objective as a regularization constraint. Let the extrinsic parameters estimated for frame t be $\left(R_{t},t_{t}\right)$, the spatiotemporal consistency loss is defined as:


$$\begin{array}{c} L_{temp}=\sum t={\text{2}}^{T}\left(|Rt-Rt-\text{1}{|}_{F}^{\text{2}}+\lambda_{t}|tt-tt-\text{1}{|}_{\text{2}}^{\text{2}}\right) \end{array}$$ (14)

where $∥\cdot {∥}_{\text{F}}$ denotes the Frobenius norm, and $\lambda_{\text{t}}>\text{0}$ is the translation consistency weight coefficient that balances the translational variation term with the rotational variation term in adjacent-frame extrinsic-parameter estimation. This temporal consistency term penalizes abrupt changes in extrinsic parameters between adjacent frames, thereby suppressing random fluctuations in single-frame estimation.

Integrating the confidence-weighted geometric reprojection error and spatiotemporal consistency constraints, the joint optimization objective function is:


$$\begin{array}{c} L_{total}=E(R,t)+\alpha L_{temp}+\beta L_{reg} \end{array}$$ (15)

where $L_{\text{reg}}$ is the rotation matrix orthogonality regularization term, and α, β are balancing coefficients. E(R,t) denotes the confidence-weighted geometric reprojection error over the established cross-modal correspondences. In this term, each reprojection residual is weighted by its corresponding confidence score ${\text{w}}_{\text{ij}}$, so that high-confidence correspondences contribute more strongly to the geometric reprojection term, while low-confidence correspondences are down-weighted before optimization. $L_{\text{temp}}$ constrains the temporal consistency of extrinsic parameters across adjacent frames and suppresses abrupt frame-to-frame fluctuations in rotation and translation. $L_{\text{reg}}$ encourages the estimated rotation matrix to satisfy the orthogonality constraint of SO(3), thereby improving the numerical stability of the solution. The above objective is formulated as a nonlinear least-squares problem and is iteratively solved using a Levenberg–Marquardt (LM) strategy [26]. In implementation, the optimization module is realized with the Ceres Solver framework, which provides efficient numerical support for parameter estimation.

**Fig 3:**
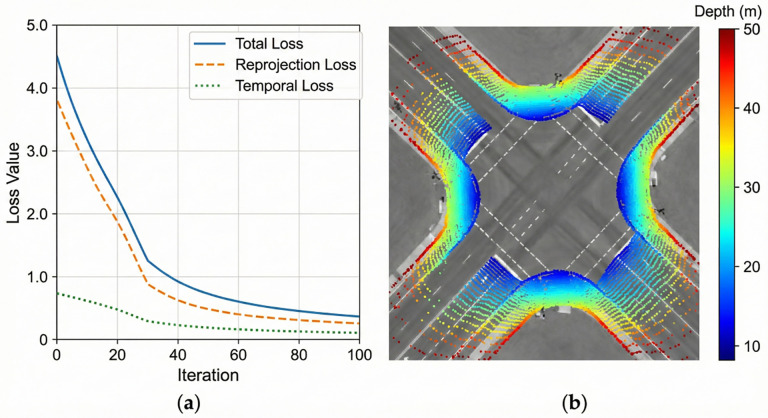
Joint Optimization Convergence Process and Calibration Result Visualization.

As shown in Fig 3, the optimization process visualization demonstrates the trend of reprojection error with iteration number and the final point cloud-image alignment effect.

Wide-area monitoring scenarios typically deploy multiple groups of LiDAR-camera systems to achieve full coverage [27]. Let the system contain K groups of sensor pairs, with extrinsic parameters $(R_{k},tk)k={\text{1}}^{K}$ for each group. Overlapping field-of-view regions exist between adjacent sensor pairs, and co-visible features in the overlap region can establish cross-constraints:


$$\begin{array}{c} L_{cross}=\sum (k,l)\in N\sum_{P\in O_{kl}}|\pi_{k}(T_{k}P)-\pi_{l}(T_{l}P){|}_{\text{2}}^{\text{2}} \end{array}$$ (16)

where N is the index set of adjacent sensor pairs, $O_{kl}$ is the set of co-visible points between the k -th and l -th sensor groups, and $T_{k}=[R_{k}|t_{k}]$ is the homogeneous transformation matrix. This constraint ensures that calibration results of adjacent sensor pairs mutually verify each other in the overlap region, improving overall consistency [28].

### 2.4. Experimental design and algorithm implementation

The experimental data were collected from typical urban intersection scenes. The roadside sensing system is composed of livox Avia solid-state lidar (non repetitive scanning mode, field angle 70.4 ° × 77.2 °) and Hikvision industrial camera (resolution 1920 × 1080, frame rate 30fps) [29]. The sensor is installed on the top of a 6m high rod with a top view angle of about 25 °, and the monitoring range covers an area of 50m × 30m. The collection period includes morning peak, flat peak and night, and about 2 hours of valid data are recorded in total.

The core algorithm is implemented based on the PyTorch framework. The Open3D library is used for point cloud processing, while the Ceres Solver is used to perform nonlinear optimization [30]. As shown in algorithm 1, the pseudo code implementation of target free collaborative calibration is given.

Algorithm 1. Roadside LiDAR-Camera Target-free Collaborative Calibration Algorithm

Line Pseudocode

1  Input: Point cloud sequence $Ptt={\text{1}}^{T}$, image sequence $Itt={\text{1}}^{T}$, camera intrinsics K_c_

2  Output: Extrinsic parameters$(R^{,}t^{)}$

3  for t=1 to T do

4  $F_{P}^{t}\leftarrow$ExtractPointFeature($P_{t}$)

5  $F_{I}^{t}\leftarrow$ExtractImageFeature($I_{t}$)

6  $A^{t}\leftarrow$CoarseMatching($F_{P}^{t}$, $F_{I}^{t}$)

7  $M_{t}\leftarrow$FineMatching($A^{t}$, $P_{t}$, $I_{t}$)

8  $(R_{t},t_{t})\leftarrow$PnPRANSAC($M_{t}$, K)

9  end for

10  $(R^{,}t^{)}\leftarrow$TemporalOptimization($\left(R_{t},t_{t}\right)$, $M_{t}$)

11  return$(R^{,}t^{)}$

The feature extraction network is pre trained on the synthetic data set, and the cross modal features are aligned in the embedded space by using the comparative learning strategy [31]. The training batch size is set to 16, and the initial learning rate is 0.001, which is adjusted by cosine annealing strategy. The single frame processing time in the reasoning phase is about 85ms (NVIDIA RTX 3080), which meets the requirements of quasi real-time applications.

The following quantitative indicators are used for calibration accuracy evaluation [32]:

(1) Rotation Error (RE): Angular deviation between estimated rotation and ground truth, in degrees (°).
(2) Translation Error (TE): Euclidean distance between estimated translation and ground truth, in centimeters (cm).
(3) Reprojection error (RPE): the average pixel offset of all interior points, in pixels (PX).

**Fig 4:**
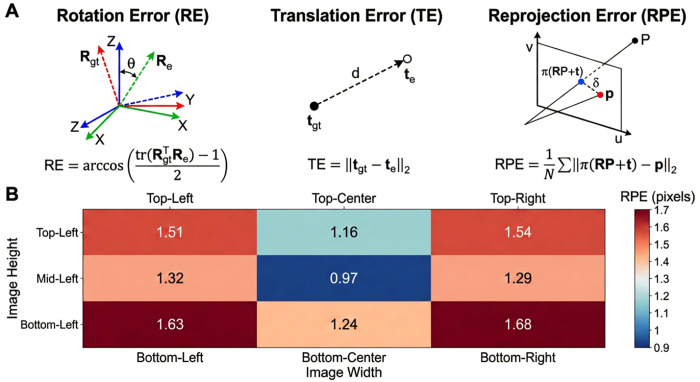
Calibration Error Evaluation Metrics and Spatial Distribution Analysis.

As shown in Fig 4, the calculation process of evaluation index and the visualization of spatial distribution of calibration error.

Additionally, this paper evaluates algorithm robustness metrics, including convergence success rate (proportion of optimization completed within a given threshold) and iteration number distribution [33]. The performance advantages of the proposed method are comprehensively verified by comparing with the traditional method based on calibration plate [34] and other non target methods [35].

### 2.5. Field deployment permission statement

The roadside sensing system used in this study was deployed on a controlled testbed located within the campus of Anhui Sanlian University. The experimental site access was approved by the Campus Facilities Management Office of Anhui Sanlian University, which is the responsible authority for infrastructure management and on-campus road usage. The experiments were conducted on internal campus roads that are open to pedestrians and vehicles, and no public highway, restricted traffic area, or government-managed road infrastructure was involved. Therefore, no additional municipal or governmental field permits were required. Furthermore, the study did not involve human subjects, personal identity information collection, or any form of privacy-sensitive data acquisition.

## 3. Results

This chapter carries out systematic experimental verification based on the method described in Chapter 2, and comprehensively evaluates the performance of the proposed method from the dimensions of calibration accuracy, algorithm robustness, and computational efficiency. The experimental data comes from the actual collection of the roadside perception system at urban intersections. The test scenarios cover different periods of time, different lighting and different traffic flow density conditions.

### 3.1. Experimental dataset and baseline methods

The experiment uses self built roadside perception data set for verification. The data set contains 12 sets of different scene sequences, each of which lasts about 10 minutes, accumulatively collecting 18000 LIDAR point cloud frames and 18000 synchronous images. Scene types include urban trunk road intersections (4 groups), secondary trunk road intersections (4 groups), T-junctions (2 groups) and roundabouts (2 groups). The data collection period covers morning peak (7:30–9:00), flat peak (14:00–16:00), evening peak (17:30–19:00) and night (21:00–22:30), to fully test the adaptability of the algorithm under different traffic flow density and lighting conditions.

**Fig 5:**
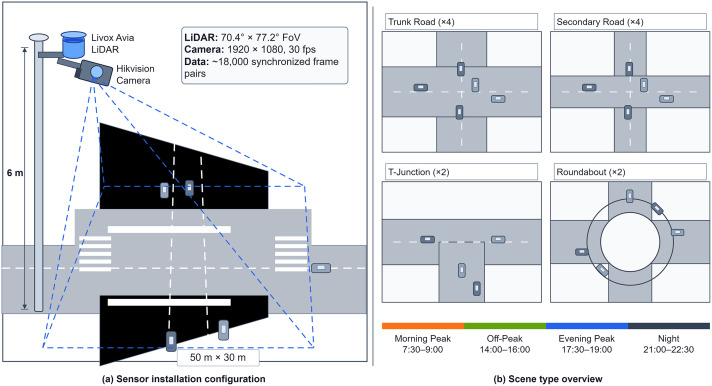
Experimental Setup and Representative Synchronized LiDAR-Camera Data Samples from Different Scene Types.

As shown in Fig 5, the roadside sensing system installation setup and representative data samples from different scene types are presented. Fig 5(a) illustrates the sensor mounting configuration on the 6-meter pole, including the relative positions of the Livox Avia LiDAR and Hikvision industrial camera with a downward viewing angle of approximately 25°. Fig 5(b)-Fig 5(e) display synchronized LiDAR point cloud and camera image pairs from four representative scene types: urban trunk road intersection, secondary trunk road intersection, T-junction, and roundabout, respectively. The point clouds are color-coded by height to visualize the three-dimensional spatial structure. It can be observed that the scenes exhibit varying levels of geometric complexity, traffic density, and structural feature richness, which collectively provide a comprehensive testbed for evaluating the proposed calibration method.

In order to obtain the calibration true value, the joint measurement method of high-precision rtk-gnss and total station is used to calibrate the external parameters of the sensor, and the measurement accuracy is better than 2 mm (translation) and 0.01 ° (rotation).

The baseline methods used for comparison include:

(1) CalibNet, an end-to-end deep learning-based calibration network;
(2) CorrI2P, an image–point cloud registration method based on dense correspondence;
(3) CoFiI2P, a coarse-to-fine correspondence-based registration method;
(4) LCCNet, a lightweight LiDAR-camera calibration network; and
(5) the traditional checkerboard-based calibration method as a reference.

All comparison methods were tested on the same hardware platform (NVIDIA RTX 3080 GPU, Intel i7-12700k CPU) using the code published by the original author.

### 3.2. Calibration accuracy evaluation

As shown in Table 2, the calibration accuracy comparison results of this method and the existing representative methods on the overall data set. The evaluation indexes include mean RE, mean TE, mean RPE and standard deviation.

**Table 2:** Calibration accuracy comparison of different methods.

Method	Mean RE (°)	Std RE (°)	Mean TE (cm)	Std TE (cm)	Mean RPE (px)	Std RPE (px)
Checkerboard	0.127	0.031	1.84	0.42	0.89	0.21
CalibNet	0.386	0.092	4.73	1.15	2.34	0.67
CorrI2P	0.294	0.078	3.65	0.94	1.87	0.52
CoFiI2P	0.231	0.064	2.91	0.73	1.52	0.41
LCCNet	0.312	0.085	3.89	1.02	1.96	0.58
Proposed Method	0.185	0.048	2.36	0.57	1.24	0.33

The experimental data show that the proposed method achieves the optimal results on the three core indicators. Compared with CoFiI2P, the average rotation error of this method is reduced by 19.9% (from 0.231 ° to 0.185 °), the average translation error is reduced by 18.9% (from 2.91 cm to 2.36 cm), and the average reprojection error is reduced by 18.4% (from 1.52px to 1.24px). It is worth noting that the standard deviation of each index of this method is also significantly smaller than that of the comparison method, which shows that the confidence guided matching strategy and spatio-temporal constraint optimization mechanism effectively improve the stability of calibration results. Although there is still a certain gap with the traditional method based on calibration board, considering that the target free method does not need manual intervention and can be automatically executed online, this accuracy level fully meets the application requirements of roadside traffic monitoring.

**Fig 6:**
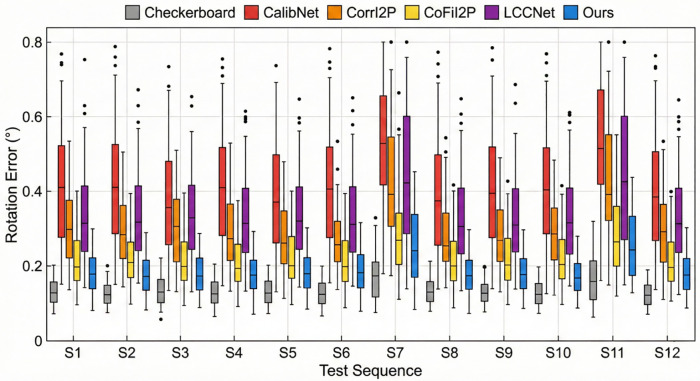
Box Plot Comparison of Rotation Errors Across Test Sequences.

As shown in Fig 6, the box diagram of rotation error distribution of different methods on each test sequence.

It can be observed from the box plot that the proposed method maintains a low median error and a narrow interquartile range in all test sequences. Sequences S7 (night scene) and S11 (roundabout) are more challenging for all methods. CalibNet and LCCNet show significant error increase in these two groups of data, while the performance decline of this method is relatively limited. This phenomenon verifies the robustness of hierarchical feature extraction strategy in weak texture and complex geometric scenes.

### 3.3. Performance analysis under different scenario conditions

The roadside sensing system needs to cope with changing environmental conditions. In this section, the performance of the method is analyzed from the three dimensions of light change, traffic flow density and sensor field position.

#### 3.3.1. Lighting Condition Impact.

As shown in Table 3, the calibration accuracy of this method is compared with CoFiI2P under different lighting conditions. The lighting conditions are divided into four categories according to the acquisition time: strong light (sunny noon), normal light (cloudy day or morning and evening), weak light (street lights at night), and extremely weak light (areas without street lights at night).

**Table 3:** Calibration accuracy comparison under different lighting conditions.

Lighting Condition	Method	Mean RE (°)	Mean TE (cm)	Mean RPE (px)
Strong Light	CoFiI2P	0.218	2.76	1.43
Strong Light	Proposed Method	0.174	2.21	1.16
Normal Light	CoFiI2P	0.209	2.64	1.38
Normal Light	Proposed Method	0.168	2.14	1.12
Weak Light	CoFiI2P	0.267	3.34	1.79
Weak Light	Proposed Method	0.203	2.58	1.38
Very Weak Light	CoFiI2P	0.341	4.28	2.31
Very Weak Light	Proposed Method	0.248	3.12	1.67

The data show that the calibration accuracy of both methods decreases with the deterioration of lighting conditions, but the decline of this method is more gentle. Under the condition of extremely weak light, the rotation error and translation error of this method are reduced by 27.3% and 27.1% respectively compared with CoFiI2P. This advantage is mainly due to the semantic understanding ability of MobileSAM segmentation network for low contrast images and the smoothing effect of spatio-temporal constraints on single frame noise.

**Fig 7:**
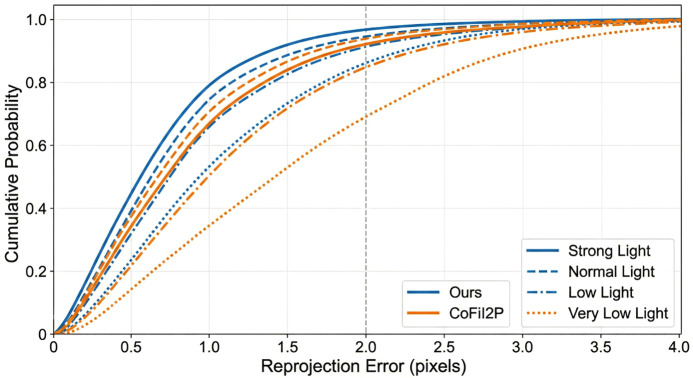
Reprojection Error CDF Curves Under Different Lighting Conditions.

As shown in Fig 7, cumulative distribution function (CDF) curves of reprojection error under different lighting conditions.

The CDF curves intuitively show error distribution characteristics. Using 2 pixels reprojection error as a threshold, the proposed method has 96.3% of matched points meeting the requirement under normal light conditions, while CoFiI2P has 89.7%; under very weak light conditions, the proposed method still has 82.1% of points meeting the threshold, while CoFiI2P drops to 68.4%.

#### 3.3.2. Traffic flow density impact.

**Fig 8:**
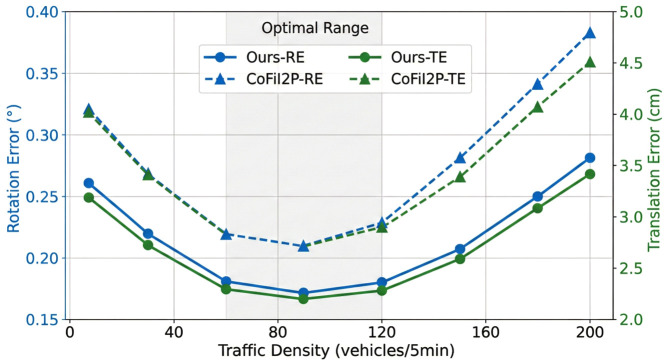
Relationship Curve Between Traffic Density and Calibration Error.

Traffic flow density directly affects the quantity and distribution of available features in the scene. As shown in Fig 8, trends of calibration error under different traffic flow densities.

Experimental results reveal an interesting phenomenon: calibration error shows a non-monotonic relationship with traffic flow density. When flow density is extremely low (<20 vehicles/5 minutes), there are insufficient dynamic features in the scene for matching, resulting in relatively high errors; when flow density is at medium level (60–120 vehicles/5 minutes), features such as vehicle contours and lane markings are abundant with limited occlusion, resulting in lowest errors; when flow density is too high (>160 vehicles/5 minutes), severe occlusion and feature confusion lead to error rebound. The proposed method outperforms CoFiI2P across all density ranges, with especially pronounced advantages in high-density scenarios, benefiting from the effective suppression of low-quality matches by the confidence weighting mechanism.

#### 3.3.3 Field-of-view spatial position impact.

Wide-area calibration needs to focus on accuracy distribution in different regions of the sensor field of view. Dividing images into 3 × 3 for a total of 9 regions, average reprojection errors for each region are separately calculated. As shown in Table 4.

**Table 4:** Reprojection error distribution in different image regions (Unit: pixels).

Region	Top-Left	Top-Center	Top-Right	Mid-Left	Center	Mid-Right	Bottom-Left	Bottom-Center	Bottom-Right
CoFiI2P	1.89	1.42	1.94	1.61	1.18	1.58	2.07	1.53	2.12
Proposed Method	1.51	1.16	1.54	1.32	0.97	1.29	1.63	1.24	1.68
Improvement	20.10%	18.30%	20.60%	18.00%	17.80%	18.40%	21.30%	18.90%	20.80%

Data show that both methods exhibit a spatial distribution pattern of low error in the center region and high error in edge regions, closely related to camera distortion and spatial non-uniformity of LiDAR point cloud density. The proposed method has higher improvement ratios in edge regions (especially the four corners), averaging 20.7%, indicating that the multi-scale feature fusion strategy effectively improves feature representation capability in edge regions.

### 3.4. Ablation experiments

To verify the contribution of each module, ablation experiments are designed to progressively remove or replace key components. As shown in Table 5.

**Table 5:** Ablation experiment results.

Configuration	Feature Extraction	Matching Strategy	Spatiotemporal Constraint	Mean RE (°)	Mean TE (cm)	Mean RPE (px)
A	PointNet++	Dense Matching	No	0.312	3.87	1.96
B	Voxel+Transformer	Dense Matching	No	0.268	3.31	1.71
C	Voxel+Transformer	Coarse-to-fine	No	0.234	2.89	1.49
D	Voxel+Transformer	Confidence-guided Coarse-to-fine	No	0.212	2.63	1.36
E (Complete)	Voxel+Transformer	Confidence-guided Coarse-to-fine	Yes	0.185	2.36	1.24

Ablation experiments clearly demonstrate the gain effect of each module. Configuration A is the baseline model, using traditional PointNet++ feature extraction and dense matching strategy. After Configuration B introduces voxelization and Transformer structure, rotation error decreases by 14.1%, validating the promotional effect of global attention mechanism on point cloud feature learning. Configuration C replaces dense matching with coarse-to-fine strategy, with error further decreasing by 12.7%, indicating hierarchical matching effectively filters low-quality correspondences. After Configuration D adds confidence weighting mechanism, error decreases by 9.4%, confirming the effectiveness of this mechanism for outlier suppression. After the introduction of space-time constraints in Configuration E, the error is reduced by an additional 12.7%, which is particularly significant in night and high occlusion scenes.

**Fig 9:**
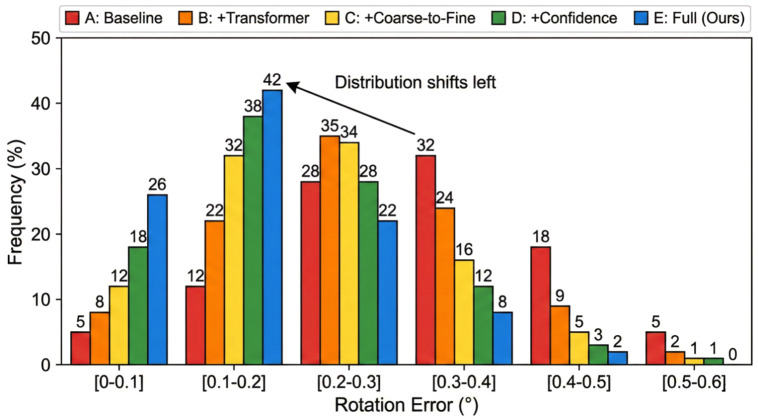
Ablation experiment error distribution histogram.

As shown in Fig 9, the error distribution histogram of each configuration of ablation experiment.

### 3.5. Temporal consistency analysis

**Fig 10:**
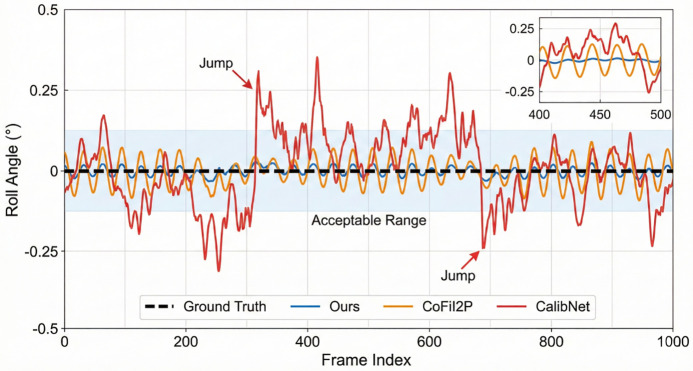
Temporal Curve of Rotation Parameter Estimation Over Consecutive Frames.

The timing stability of calibration results is very important for actual deployment. As shown in Fig 10, the time-varying curve of the rotation parameters estimated by each method (taking Euler angle roll as an example) over 1000 consecutive frames.

From the temporal curves, it can be observed that CalibNet’s estimation results exhibit large fluctuations, with obvious jumps near frames 312 and 687, related to its single-frame regression strategy lacking temporal constraints. The fluctuation amplitude of CoFiI2P is in the middle, and it is generally stable, but there is still periodic jitter. The method in this paper benefits from the constraint of spatio-temporal consistency loss. The curve is the smoothest, and the deviation from the true value is kept within ± 0.08 °. Quantitative statistics show that the standard deviation of the inter-frame rotation parameters of this method is 0.023 °, which is 56% of CoFiI2P (0.041 °) and 29% of CalibNet (0.078 °), respectively.

### 3.6. Computational efficiency analysis

Real time performance is the key index of roadside sensing system. As shown in Table 6, the calculation time statistics of each method.

**Table 6:** Computational efficiency comparison.

Method	Feature Extraction (ms)	Matching (ms)	Optimization (ms)	Total Time (ms)	Frame Rate (FPS)
CalibNet	42.3	–	8.1	50.4	19.8
CorrI2P	38.7	24.6	12.3	75.6	13.2
CoFiI2P	41.2	31.8	14.5	87.5	11.4
LCCNet	35.6	–	6.8	42.4	23.6
Proposed Method	47.8	26.4	10.9	85.1	11.8

The total time of single frame is 85.1ms, and the corresponding frame rate is about 11.8fps. The feature extraction phase takes 47.8ms, which is slightly higher than the comparison method, mainly due to the computational overhead of transformer structure; The matching phase takes 26.4ms, thanks to the coarse to fine strategy which reduces the candidate size of fine-grained matching; The optimization phase takes 10.9ms, and the amount of additional calculation introduced by space-time constraints is limited. Considering the accuracy and efficiency, the proposed method achieves significant accuracy improvement while maintaining the frame rate similar to CoFiI2P. For the 30fps video stream, the interlaced processing strategy can be used to complete the online calibration under the premise of limited timeliness.

**Fig 11:**
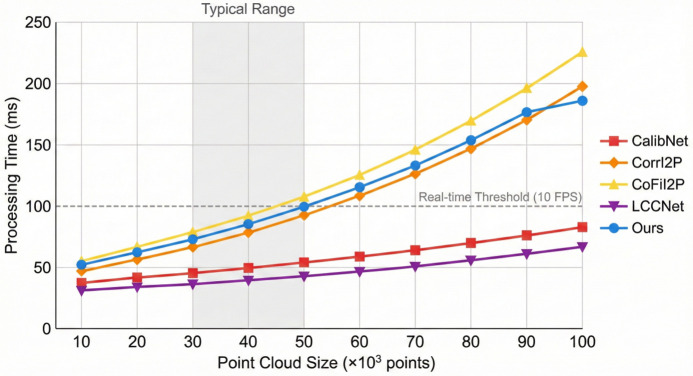
Relationship curve between point cloud scale and computation time.

As shown in Fig 11, time consumption variation curves for each method under different input point cloud scales.

When point cloud scale increases from 10k to 100k, the proposed method’s time consumption increases from 52ms to 187ms, a growth rate of 3.6 times. Voxelization preprocessing effectively controls the Transformer’s input sequence length, avoiding computational explosion caused by quadratic complexity. Under typical roadside LiDAR configuration (approximately 30k-50k points/frame), the proposed method can stably maintain 80–110ms processing time.

### 3.7. Robustness verification

To evaluate the method’s tolerance to extreme conditions, three groups of robustness tests are designed: (1) artificially adding different proportions of outlier interference; (2) simulating sensor installation offset; (3) introducing timestamp synchronization error.

**Fig 12:**
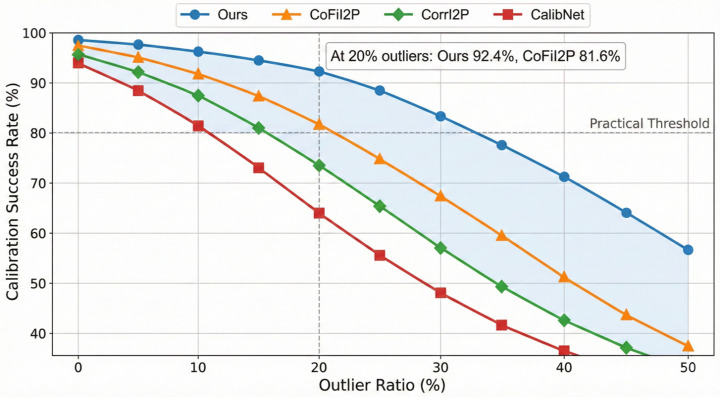
Relationship curve between outlier ratio and calibration success rate.

As shown in Fig 12, calibration success rate curves under different outlier ratios for each method. Success criterion is defined as rotation error <0.5° and translation error <5 cm.

When outlier ratio is 20%, the proposed method’s success rate still maintains 92.4%, CoFiI2P is 81.6%, CorrI2P is 73.8%, and CalibNet is 64.2%. When outlier ratio reaches 40%, the proposed method’s success rate decreases to 71.3%, still the highest among all methods. The synergistic effect of confidence weighting mechanism and RANSAC significantly improves outlier tolerance.

In the sensor installation offset test, random perturbations (rotation ±5°, translation ±20 cm) are artificially superimposed on the ground truth extrinsic parameters to simulate initial calibration inaccuracy. The proposed method can still converge to the correct solution in 94.7% of cases under this condition, while CalibNet, lacking iterative optimization mechanism, has only 67.3% convergence rate. In the timestamp synchronization test, introducing random time offsets of ±50ms, the proposed method’s average rotation error increases from 0.185° to 0.227° (increase of 22.7%), and translation error increases from 2.36 cm to 2.89 cm (increase of 22.5%), with performance degradation within acceptable range.

**Fig 13:**
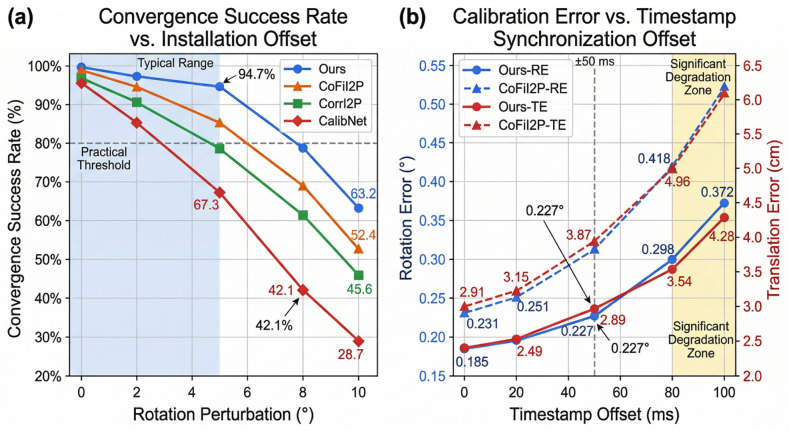
Robustness test results under sensor installation offset and timestamp synchronization error.

As shown in Fig 13, the detailed robustness test results under sensor installation offset and timestamp synchronization error are presented. Fig 13(a) displays the convergence success rate of each method under different levels of initial installation offset, where the horizontal axis represents the magnitude of rotation perturbation (ranging from ±1° to ±10°) and the vertical axis represents the convergence success rate. It can be observed that the proposed method maintains a success rate above 90% when the rotation perturbation is within ±5°, and still achieves 78.6% at ±8°, significantly outperforming CalibNet (42.1%) and CorrI2P (61.3%) under the same condition. Fig 13(b) shows the calibration error variation curves of the proposed method and CoFiI2P under different timestamp synchronization offsets (ranging from ±10ms to ±100ms). When the synchronization offset is within ±50ms, the proposed method’s rotation error remains below 0.23°, benefiting from the smoothing effect of the spatiotemporal consistency constraint. When the offset exceeds ±80ms, both methods show significant accuracy degradation, indicating that hardware-level time synchronization is still necessary for practical deployment.

Based on the above experimental results, the proposed roadside lidar camera wide area collaborative calibration method shows better performance than the existing methods in terms of calibration accuracy, environmental adaptability, time sequence stability and robustness, and verifies the effectiveness of three technological innovations: hierarchical feature extraction, confidence guided matching and spatio-temporal constraint optimization.

## 4. Discussion

### 4.1. Method performance analysis and comparison

The proposed roadside lidar camera wide area collaborative calibration method shows significant performance advantages in a number of evaluation indicators, which is closely related to the three core technological innovations integrated into the method design. The experimental data show that the hierarchical feature extraction strategy effectively captures the local geometric details and long-range spatial dependencies in the point cloud data through the synergy of voxel coding and transformer’s global attention mechanism, which makes the feature expression achieve a good balance between semantic richness and geometric accuracy; The confidence guided coarse to fine matching strategy is established through two-stage progressive correspondence, which not only ensures the matching recall rate, but also significantly inhibits the introduction of error correspondence. The error reduction from configuration C to configuration D in Ablation Experiment (9.4%) fully verifies the effectiveness of the mechanism; The spatio-temporal constraint optimization module uses the consistency prior of external parameters between consecutive frames to smooth the random noise in single frame estimation, so as to control the inter-frame standard deviation of calibration results at a low level of 0.023°.The ablation trend further indicates that the optimization gain arises from the coupling sequence among feature extraction, confidence-guided matching, and temporal refinement. Configuration D first filters unreliable cross-modal correspondences through confidence weighting, and Configuration E then applies fixed-sensor temporal consistency to the filtered correspondence set. This processing order reduces the propagation of unstable matches caused by occlusion, weak illumination, and dense traffic flow. The reduction from Configuration D to Configuration E therefore reflects the effect of integrating confidence-weighted target-free matching with roadside temporal stability. This architectural arrangement is particularly suitable for fixed roadside deployments because the extrinsic parameters are expected to remain stable across adjacent frames, while observed traffic objects vary dynamically.

As shown in Table 7, the comprehensive comparison between the proposed method and the existing representative non target calibration methods in terms of key performance dimensions comprehensively presents the advantages and disadvantages of each method in terms of accuracy, robustness, efficiency and applicable scenarios.

**Table 7:** Comprehensive performance comparison of target-free calibration methods.

Evaluation Dimension	Metric	CalibNet	CorrI2P	CoFiI2P	LCCNet	Proposed Method
Accuracy	Rotation Error (°)	0.386	0.294	0.231	0.312	0.185
Accuracy	Translation Error (cm)	4.73	3.65	2.91	3.89	2.36
Robustness	20% Outlier Success Rate	64.20%	73.80%	81.60%	68.40%	92.40%
Robustness	Very Weak Light Error Increase	89.40%	62.30%	47.40%	71.80%	34.10%
Efficiency	Single Frame Time (ms)	50.4	75.6	87.5	42.4	85.1
Efficiency	Parameters (M)	12.8	8.6	11.2	5.4	9.7
Applicability	No Initial Calibration Required	No	Yes	Yes	No	Yes
Applicability	Supports Online Update	No	Yes	Yes	No	Yes

From Table 7, it can be clearly observed that the proposed method’s performance in accuracy and robustness dimensions is significantly better than all comparison methods. Although it is slightly inferior to LCCNet and CalibNet lightweight network designs in computational efficiency, considering that the 85.1ms single-frame time can already meet near real-time application requirements (frame-skipping processing can achieve approximately 6FPS online calibration frequency), this efficiency cost is acceptable. It is particularly noteworthy that the proposed method’s error increase under very weak light conditions is only 34.1%, far lower than the 47.4%−89.4% increase range of other methods. This significant advantage stems from MobileSAM semantic segmentation network’s robust understanding capability for low-contrast images and the effective suppression of single-frame noise by spatiotemporal constraints. Although CalibNet and LCCNet have higher computational efficiency, due to adopting end-to-end regression strategy lacking explicit geometric constraint modeling, their accuracy and robustness in complex scenarios are difficult to reach satisfactory levels, which also confirms the rationality of adhering to the “feature matching + geometric optimization” technical route in the method design of this paper.

### 4.2. Limitations and improvement directions

Although the proposed method has achieved encouraging results in experimental verification, there are still several limitations worthy of attention that need to be addressed in future research. From the algorithm level, the current feature extraction network’s generalization capability still has room for improvement, and performance degradation may occur in extreme scenarios not covered by training data (such as heavy rain, dense fog, snow-covered road surfaces). Domain adaptive learning or self-supervised pre-training strategies can be introduced in the future to enhance the model’s adaptability to out-of-distribution data; although the coarse-to-fine strategy in the matching stage effectively improves correspondence quality, the cascaded structure of two stages also makes errors from the coarse matching stage potentially propagate downstream. Exploring end-to-end differentiable joint matching-optimization frameworks is a worthwhile research direction. From the system level, this paper’s experiments are mainly based on fixed-installation roadside sensor configurations. For temporary deployment or mobile platform calibration scenarios, relative positions between sensors may drift slowly over time. The current spatiotemporal constraint model assumes extrinsic parameters remain constant within short time windows and does not yet have adaptive tracking capability for such dynamic changes; additionally, computational complexity of multi-sensor collaborative calibration grows super-linearly with the number of sensors. How to design more efficient distributed optimization algorithms while ensuring accuracy is also an engineering problem that must be solved to promote this technology toward large-scale deployment.

From a more macro application perspective, the value of this method is not only reflected in the improvement of calibration accuracy, but also in the possibility of Paradigm Innovation for the deployment and operation mode of roadside sensing system due to its aimless, online and automated technical characteristics. The traditional method based on calibration board needs manual intervention, which will cause traffic interruption and is difficult to adapt to the long-term drift of sensor attitude. However, this method can use the traffic flow itself as a natural calibration signal source to continuously monitor and automatically update the external parameters during the normal operation of the system. This ability has important practical significance for the construction of intelligent transportation infrastructure with high reliability and low maintenance cost. Future research will focus on solving the above limitations, and explore the extension of this method to the dynamic joint calibration in the vehicle road coordination scene, in order to provide more perfect perception infrastructure support technology for the next generation of intelligent transportation system.

## 5. Conclusion

To address the practical demand for LiDAR-camera extrinsic calibration in roadside traffic monitoring, this paper proposes a target-free wide-area collaborative calibration method. This method achieves the goal of automatically establishing cross modal correspondence and accurately estimating sensor external parameters in natural traffic scene through three core technological innovations, namely hierarchical feature extraction, confidence guided matching and spatio-temporal constraint optimization. The experimental results show that the average rotation error of this method is 0.185°, the average translation error is 2.36 cm, and the average reprojection error is 1.24 pixels, which is reduced by 19.9%, 18.9% and 18.4% respectively compared with the existing optimal target free method CoFiI2P, showing more robust performance under challenging conditions such as extremely weak light, high occlusion, and large outlier ratio. Ablation experiments verified the effectiveness of each module design, in which voxel transformer feature coding contributed 14.1% error reduction, confidence weighted mechanism contributed 9.4% error reduction, and spatio-temporal consistency constraint contributed 12.7% error reduction. The calculation efficiency of single frame processing time-consuming 85.1ms can meet the requirements of quasi real-time applications, and the calibration success rate of 92.4% under a 20% outlier ratio demonstrates the practical robustness of the method.

The main contribution of this paper is to establish a complete set of roadside lidar camera non target calibration technology framework, which provides a new technical scheme for the deployment and operation of the perception system of intelligent transportation infrastructure. This method gets rid of the dependence on manual calibration board, and can realize online automatic calibration by using the characteristics of traffic flow. It has the advantages of convenient deployment, low maintenance cost and sustainable self calibration. The current method still has room for improvement in extreme weather adaptability and multi-sensor collaborative optimization efficiency. The subsequent research will introduce domain adaptive learning to improve the generalization ability of the model, and explore the dynamic joint calibration mechanism for vehicle road collaborative scenes, so as to further expand the application boundary and practical value of the method.
